# {4,4′-Dibromo-2,2′-[ethane-1,2-diylbis(nitrilo­methyl­idyne)]diphenolato}copper(II)

**DOI:** 10.1107/S1600536809024015

**Published:** 2009-07-11

**Authors:** Qing-Fan Xie, Yan-Min Chen, Miao-Ling Huang

**Affiliations:** a Department of Chemistry and Science of Life, Quanzhou Normal University, Fujian 362000, People’s Republic of China

## Abstract

In the title compound, [Cu(C_16_H_12_Br_2_N_2_O_2_)], the Cu^II^ atom is coordinated in a slightly distorted square-planar geometry by two O and two N atoms of the tetra­dentate dianionic 4,4′-dibromo-2,2′-[ethane-1,2-diylbis(nitrilo­methyl­idyne)]diphen­olate Schiff base ligand.

## Related literature

For background to complexes of Schiff bases, see: Arnold *et al.* (1998 ▶); Jabri *et al.* (1995 ▶); Jiang *et al.* (2003 ▶). For a related structure, see: Feng *et al.* (2007 ▶).
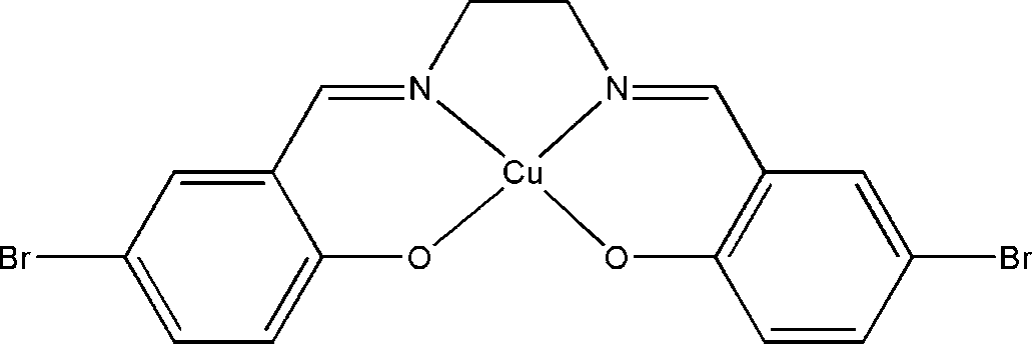


## Experimental

### 

#### Crystal data

[Cu(C_16_H_12_Br_2_N_2_O_2_)]
*M* *_r_* = 487.68
Triclinic, 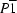

*a* = 8.2848 (4) Å
*b* = 9.6302 (5) Å
*c* = 10.9984 (6) Å
α = 115.601 (6)°
β = 92.866 (4)°
γ = 101.527 (5)°
*V* = 766.10 (7) Å^3^
*Z* = 2
Mo *K*α radiation
μ = 6.65 mm^−1^
*T* = 173 K
0.4 × 0.1 × 0.1 mm

#### Data collection

Oxford Diffraction Xcalibur diffractometer with a Sapphire CCD detector
Absorption correction: multi-scan (*SADABS*; Sheldrick, 1996 ▶) *T*_min_ = 0.715, *T*_max_ = 1 (expected range = 0.368–0.514)
6303 measured reflections
2641 independent reflections
2128 reflections with *I* > 2σ(*I*)
*R*_int_ = 0.030

#### Refinement

*R*[*F* ^2^ > 2σ(*F* ^2^)] = 0.027
*wR*(*F* ^2^) = 0.060
*S* = 1.01
2641 reflections
208 parameters
H-atom parameters constrained
Δρ_max_ = 0.50 e Å^−3^
Δρ_min_ = −0.35 e Å^−3^

### 

Data collection: *SMART* (Bruker, 2001 ▶); cell refinement: *SAINT* (Bruker, 2003 ▶); data reduction: *SAINT*; program(s) used to solve structure: *SHELXS97* (Sheldrick, 2008 ▶); program(s) used to refine structure: *SHELXL97* (Sheldrick, 2008 ▶); molecular graphics: *SHELXTL* (Sheldrick, 2008 ▶); software used to prepare material for publication: *SHELXTL*.

## Supplementary Material

Crystal structure: contains datablocks global, I. DOI: 10.1107/S1600536809024015/tk2481sup1.cif

Structure factors: contains datablocks I. DOI: 10.1107/S1600536809024015/tk2481Isup2.hkl

Additional supplementary materials:  crystallographic information; 3D view; checkCIF report

## Figures and Tables

**Fig. 1.**
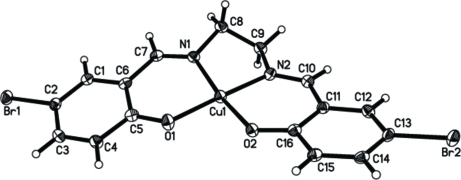
Molecular structure of (I) showing atom labelling and displacement ellipsoids at the 30% probability level.

## supplementary crystallographic information

### Comment

Schiff base complexes continue to attract attention owing to their anti-bacterial, anti-viral and other biological activities (Arnold *et al.*,  1998; Jabri *et al.*, 1995; Jiang *et al.*, 2003). In order to extend the study of these compounds, the title complex (I) was synthesized and its crystal structure determined.

The copper atom in the mononuclear complex, Fig. 1, assumes a N_2_O_2_ coordination geometry provided by the dinegative, tetradentate Schiff base ligand. The coordination  geometry approximates a square planar arrangement. The structure of (I) resembles that of N,N'-ethylene-bis(salicylaldiminato)nickel(II) (Feng *et al.*, 2007).

### Experimental

A mixture of N,N'-ethylene-bis(5-bromosalicylaldimine) (0.1 mmol), Cu(NO_3_)_2_ (0.1 mmol), DMF (10.0 ml), and ethanol (5.0 ml) was sealed in a 40 mL Teflon-lined stainless steel reactor, heated in an oven at 353 K for 72 h, and then slowly cooled to room temperature. The blue crystals were collected.

### Refinement

Carbon-bound H-atoms were placed in calculated positions with C—H = 0.93 – 0.97 Å, and were included in the refinement in the riding model approximation with *U*(H) set to 1.2*U*_eq_(C).

### Crystal data

**Table:**

[Cu(C_16_H_12_Br_2_N_2_O_2_)]	*Z* = 2
*M**_r_* = 487.68	*F*(000) = 474
Triclinic, *P*1	*D*_x_ = 2.114 Mg m^−^^3^
Hall symbol: -P 1	Mo *K*α radiation, λ = 0.71073 Å
*a* = 8.2848 (4) Å	Cell parameters from 3643 reflections
*b* = 9.6302 (5) Å	θ = 2.4–32.6°
*c* = 10.9984 (6) Å	µ = 6.65 mm^−^^1^
α = 115.601 (6)°	*T* = 173 K
β = 92.866 (4)°	Block, blue
γ = 101.527 (5)°	0.4 × 0.1 × 0.1 mm
*V* = 766.10 (7) Å^3^	

### Data collection

**Table:**

Oxford Diffraction Xcalibur diffractometer with a Sapphire CCD detector	2641 independent reflections
Radiation source: fine-focus sealed tube	2128 reflections with *I* > 2σ(*I*)
graphite	*R*_int_ = 0.030
Rotation method data acquisition using ω and φ scans	θ_max_ = 25.0°, θ_min_ = 2.4°
Absorption correction: multi-scan (*SADABS*; Sheldrick, 1996)	*h* = −9→9
*T*_min_ = 0.715, *T*_max_ = 1	*k* = −11→11
6303 measured reflections	*l* = −13→13

### Refinement

**Table:**

Refinement on *F*^2^	Primary atom site location: structure-invariant direct methods
Least-squares matrix: full	Secondary atom site location: difference Fourier map
*R*[*F*^2^ > 2σ(*F*^2^)] = 0.027	Hydrogen site location: inferred from neighbouring sites
*wR*(*F*^2^) = 0.060	H-atom parameters constrained
*S* = 1.01	*w* = 1/[σ^2^(*F*_o_^2^) + (0.0298*P*)^2^] where *P* = (*F*_o_^2^ + 2*F*_c_^2^)/3
2641 reflections	(Δ/σ)_max_ = 0.005
208 parameters	Δρ_max_ = 0.50 e Å^−^^3^
0 restraints	Δρ_min_ = −0.35 e Å^−^^3^

### Special details

**Table:**

Geometry. All e.s.d.'s (except the e.s.d. in the dihedral angle between two l.s. planes) are estimated using the full covariance matrix. The cell e.s.d.'s are taken into account individually in the estimation of e.s.d.'s in distances, angles and torsion angles; correlations between e.s.d.'s in cell parameters are only used when they are defined by crystal symmetry. An approximate (isotropic) treatment of cell e.s.d.'s is used for estimating e.s.d.'s involving l.s. planes.
Refinement. Refinement of *F*^2^ against ALL reflections. The weighted *R*-factor *wR* and goodness of fit *S* are based on *F*^2^, conventional *R*-factors *R* are based on *F*, with *F* set to zero for negative *F*^2^. The threshold expression of *F*^2^ > σ(*F*^2^) is used only for calculating *R*-factors(gt) *etc*. and is not relevant to the choice of reflections for refinement. *R*-factors based on *F*^2^ are statistically about twice as large as those based on *F*, and *R*- factors based on ALL data will be even larger.

### Fractional atomic coordinates and isotropic or equivalent isotropic displacement parameters (Å^2^)

**Table:**

	*x*	*y*	*z*	*U*_iso_*/*U*_eq_	
Cu1	0.98239 (6)	1.20034 (5)	0.06363 (4)	0.02426 (13)	
Br1	0.55269 (5)	0.80260 (4)	0.44272 (4)	0.03477 (13)	
Br2	1.24837 (5)	1.57593 (5)	−0.37940 (4)	0.03793 (13)	
O2	0.9055 (3)	1.2075 (3)	−0.1004 (2)	0.0258 (6)	
O1	0.7760 (3)	1.0630 (3)	0.0529 (2)	0.0332 (6)	
N2	1.1879 (4)	1.3606 (3)	0.1009 (3)	0.0241 (7)	
N1	1.0890 (4)	1.1769 (3)	0.2128 (3)	0.0241 (7)	
C16	0.9859 (5)	1.2886 (4)	−0.1568 (4)	0.0250 (8)	
C15	0.9173 (5)	1.2616 (4)	−0.2887 (4)	0.0273 (8)	
H15A	0.8176	1.1854	−0.3323	0.033*	
C14	0.9942 (5)	1.3449 (4)	−0.3533 (4)	0.0272 (9)	
H14A	0.9469	1.3238	−0.4399	0.033*	
C13	1.1434 (5)	1.4613 (4)	−0.2894 (4)	0.0280 (9)	
C12	1.2146 (5)	1.4889 (4)	−0.1645 (4)	0.0278 (9)	
H12A	1.3138	1.5666	−0.1229	0.033*	
C11	1.1425 (5)	1.4034 (4)	−0.0966 (4)	0.0246 (8)	
C10	1.2328 (5)	1.4321 (4)	0.0297 (4)	0.0257 (8)	
H10A	1.3328	1.5096	0.0626	0.031*	
C5	0.7334 (5)	1.0083 (4)	0.1394 (4)	0.0266 (8)	
C4	0.5657 (5)	0.9251 (4)	0.1244 (4)	0.0295 (9)	
H4A	0.4890	0.9111	0.0529	0.035*	
C3	0.5139 (5)	0.8652 (4)	0.2115 (4)	0.0281 (9)	
H3A	0.4029	0.8122	0.1992	0.034*	
C2	0.6253 (5)	0.8827 (4)	0.3184 (4)	0.0257 (8)	
C1	0.7877 (5)	0.9614 (4)	0.3382 (4)	0.0248 (8)	
H1A	0.8613	0.9728	0.4105	0.030*	
C6	0.8457 (5)	1.0256 (4)	0.2511 (4)	0.0246 (8)	
C7	1.0206 (5)	1.1058 (4)	0.2782 (4)	0.0254 (8)	
H7A	1.0876	1.1053	0.3483	0.030*	
C8	1.2681 (5)	1.2481 (4)	0.2451 (4)	0.0290 (9)	
H8A	1.3272	1.1760	0.1838	0.035*	
H8B	1.3095	1.2730	0.3381	0.035*	
C9	1.2922 (5)	1.3988 (4)	0.2276 (4)	0.0279 (8)	
H9A	1.2599	1.4812	0.3043	0.033*	
H9B	1.4083	1.4368	0.2233	0.033*	

### Atomic displacement parameters (Å^2^)

**Table:**

	*U*^11^	*U*^22^	*U*^33^	*U*^12^	*U*^13^	*U*^23^
Cu1	0.0249 (3)	0.0287 (3)	0.0179 (2)	0.00328 (19)	0.00093 (18)	0.0109 (2)
Br1	0.0388 (3)	0.0389 (2)	0.0343 (2)	0.00736 (19)	0.00758 (18)	0.0242 (2)
Br2	0.0462 (3)	0.0443 (3)	0.0355 (2)	0.0127 (2)	0.00954 (19)	0.0280 (2)
O2	0.0260 (15)	0.0306 (14)	0.0192 (13)	0.0036 (11)	0.0022 (11)	0.0112 (12)
O1	0.0318 (17)	0.0439 (16)	0.0214 (14)	−0.0038 (12)	−0.0038 (11)	0.0190 (13)
N2	0.0292 (18)	0.0242 (16)	0.0174 (16)	0.0055 (13)	0.0008 (13)	0.0087 (14)
N1	0.0229 (18)	0.0279 (17)	0.0207 (17)	0.0058 (13)	0.0009 (13)	0.0107 (14)
C16	0.028 (2)	0.026 (2)	0.022 (2)	0.0113 (17)	0.0062 (16)	0.0087 (17)
C15	0.029 (2)	0.029 (2)	0.022 (2)	0.0089 (17)	0.0008 (16)	0.0101 (17)
C14	0.031 (2)	0.033 (2)	0.021 (2)	0.0151 (18)	0.0038 (16)	0.0125 (18)
C13	0.037 (2)	0.032 (2)	0.027 (2)	0.0167 (18)	0.0134 (18)	0.0190 (18)
C12	0.025 (2)	0.031 (2)	0.025 (2)	0.0058 (17)	0.0052 (16)	0.0112 (18)
C11	0.027 (2)	0.028 (2)	0.0213 (19)	0.0092 (17)	0.0042 (16)	0.0120 (16)
C10	0.027 (2)	0.0228 (19)	0.024 (2)	0.0049 (16)	0.0027 (16)	0.0076 (17)
C5	0.030 (2)	0.026 (2)	0.020 (2)	0.0083 (17)	0.0018 (16)	0.0072 (17)
C4	0.025 (2)	0.038 (2)	0.024 (2)	0.0031 (17)	−0.0012 (16)	0.0156 (18)
C3	0.024 (2)	0.029 (2)	0.028 (2)	0.0043 (17)	0.0057 (17)	0.0107 (18)
C2	0.031 (2)	0.025 (2)	0.023 (2)	0.0081 (17)	0.0071 (17)	0.0116 (17)
C1	0.028 (2)	0.024 (2)	0.021 (2)	0.0073 (16)	0.0009 (16)	0.0083 (16)
C6	0.030 (2)	0.0209 (19)	0.020 (2)	0.0088 (16)	−0.0004 (16)	0.0063 (16)
C7	0.028 (2)	0.028 (2)	0.0187 (19)	0.0094 (17)	−0.0007 (16)	0.0084 (17)
C8	0.022 (2)	0.038 (2)	0.027 (2)	0.0051 (17)	−0.0025 (16)	0.0165 (18)
C9	0.024 (2)	0.032 (2)	0.022 (2)	0.0023 (17)	−0.0020 (16)	0.0096 (17)

### Geometric parameters (Å, °)

**Table:**

Cu1—O1	1.905 (2)	C12—H12A	0.9300
Cu1—O2	1.917 (2)	C11—C10	1.431 (5)
Cu1—N1	1.943 (3)	C10—H10A	0.9300
Cu1—N2	1.945 (3)	C5—C4	1.420 (5)
Br1—C2	1.907 (3)	C5—C6	1.432 (5)
Br2—C13	1.898 (3)	C4—C3	1.362 (5)
O2—C16	1.301 (4)	C4—H4A	0.9300
O1—C5	1.302 (4)	C3—C2	1.385 (5)
N2—C10	1.272 (4)	C3—H3A	0.9300
N2—C9	1.461 (4)	C2—C1	1.361 (5)
N1—C7	1.273 (4)	C1—C6	1.404 (5)
N1—C8	1.459 (4)	C1—H1A	0.9300
C16—C15	1.423 (5)	C6—C7	1.447 (5)
C16—C11	1.432 (5)	C7—H7A	0.9300
C15—C14	1.374 (5)	C8—C9	1.521 (5)
C15—H15A	0.9300	C8—H8A	0.9700
C14—C13	1.399 (5)	C8—H8B	0.9700
C14—H14A	0.9300	C9—H9A	0.9700
C13—C12	1.358 (5)	C9—H9B	0.9700
C12—C11	1.404 (5)		
			
O1—Cu1—O2	91.88 (10)	C11—C10—H10A	117.2
O1—Cu1—N1	92.75 (11)	O1—C5—C4	119.4 (3)
O2—Cu1—N1	170.19 (12)	O1—C5—C6	124.3 (3)
O1—Cu1—N2	171.68 (11)	C4—C5—C6	116.3 (3)
O2—Cu1—N2	92.89 (11)	C3—C4—C5	122.0 (4)
N1—Cu1—N2	83.60 (12)	C3—C4—H4A	119.0
C16—O2—Cu1	127.2 (2)	C5—C4—H4A	119.0
C5—O1—Cu1	127.5 (2)	C4—C3—C2	120.5 (4)
C10—N2—C9	120.6 (3)	C4—C3—H3A	119.8
C10—N2—Cu1	126.9 (3)	C2—C3—H3A	119.8
C9—N2—Cu1	112.5 (2)	C1—C2—C3	120.3 (3)
C7—N1—C8	119.9 (3)	C1—C2—Br1	119.0 (3)
C7—N1—Cu1	127.3 (3)	C3—C2—Br1	120.7 (3)
C8—N1—Cu1	112.8 (2)	C2—C1—C6	120.9 (3)
O2—C16—C15	118.8 (3)	C2—C1—H1A	119.5
O2—C16—C11	124.6 (3)	C6—C1—H1A	119.5
C15—C16—C11	116.6 (3)	C1—C6—C5	119.9 (3)
C14—C15—C16	121.9 (3)	C1—C6—C7	117.5 (3)
C14—C15—H15A	119.1	C5—C6—C7	122.6 (3)
C16—C15—H15A	119.1	N1—C7—C6	124.6 (3)
C15—C14—C13	120.2 (3)	N1—C7—H7A	117.7
C15—C14—H14A	119.9	C6—C7—H7A	117.7
C13—C14—H14A	119.9	N1—C8—C9	105.9 (3)
C12—C13—C14	119.8 (3)	N1—C8—H8A	110.6
C12—C13—Br2	120.3 (3)	C9—C8—H8A	110.6
C14—C13—Br2	119.9 (3)	N1—C8—H8B	110.6
C13—C12—C11	121.8 (3)	C9—C8—H8B	110.6
C13—C12—H12A	119.1	H8A—C8—H8B	108.7
C11—C12—H12A	119.1	N2—C9—C8	107.7 (3)
C12—C11—C16	119.6 (3)	N2—C9—H9A	110.2
C12—C11—C10	117.8 (3)	C8—C9—H9A	110.2
C16—C11—C10	122.5 (3)	N2—C9—H9B	110.2
N2—C10—C11	125.6 (3)	C8—C9—H9B	110.2
N2—C10—H10A	117.2	H9A—C9—H9B	108.5
